# Idiopathic Chylopericardium in a Young Adult: Diagnosis and Management

**DOI:** 10.1155/2021/5596764

**Published:** 2021-06-11

**Authors:** João Pedro E. Sant'Ana, Amanda O. Vicente, Amanda S. Pereira, Pedro V. Bertozzi, Rodrigo A. S. Sardenberg

**Affiliations:** ^1^Advanced Research Center in Medicine, School of Medicine, União das Faculdades dos Grandes Lagos (UNILAGO), São José do Rio Preto SP, Brazil; ^2^Thoracic Surgeon Hospital Alemão Oswaldo Cruz, Thoracic Surgery Departament, São Paulo (SP), Brazil

## Abstract

Primary idiopathic chylopericardium (PIC) is an uncommon cardiologic disorder; it is defined as accumulation of lymph in the pericardial sac without any know precipitating factor. A 25-year-old presented with dyspnea and chest pain for over two months. The patient underwent a chest X-ray, which revealed an enlargement of cardiac silhouette and signs of cardiac tamponade. Chest CT was performed, revealing large pericardial effusion and small pleural effusion on the right hemithorax. The patient was referred to the ICU and underwent a pericardial window through VATS, which revealed 500 ml of a milky fluid.

## 1. Introduction

Primary idiopathic chylopericardium (PIC) is an uncommon cardiologic disorder, first reported by Hasebroek et al. in 1888 [1]. The term PIC was initially used by Groves and Effler in 1954, with a case of isolated lymph accumulation in the pericardium without any other evident cause [2]. Other secondary causes may occur, such as complications due to trauma, mediastinal tumors, lymphoma, cardiothoracic surgery, mediastinal tuberculosis, radiotherapy, and subclavian vein thrombosis [3]. Congenital lymphangiomatosis or lymphangiectasia may also be a cause of chylopericardium. There are relatively few published reports of PIC, and its pathogenesis remains unknown.

The present study reports a case of PIC in a 25-year-old female patient, who remained symptomatic for two months. Because of cardiac tamponade, she was treated successfully by surgical approach.

## 2. Case Report

**Figure 1:**
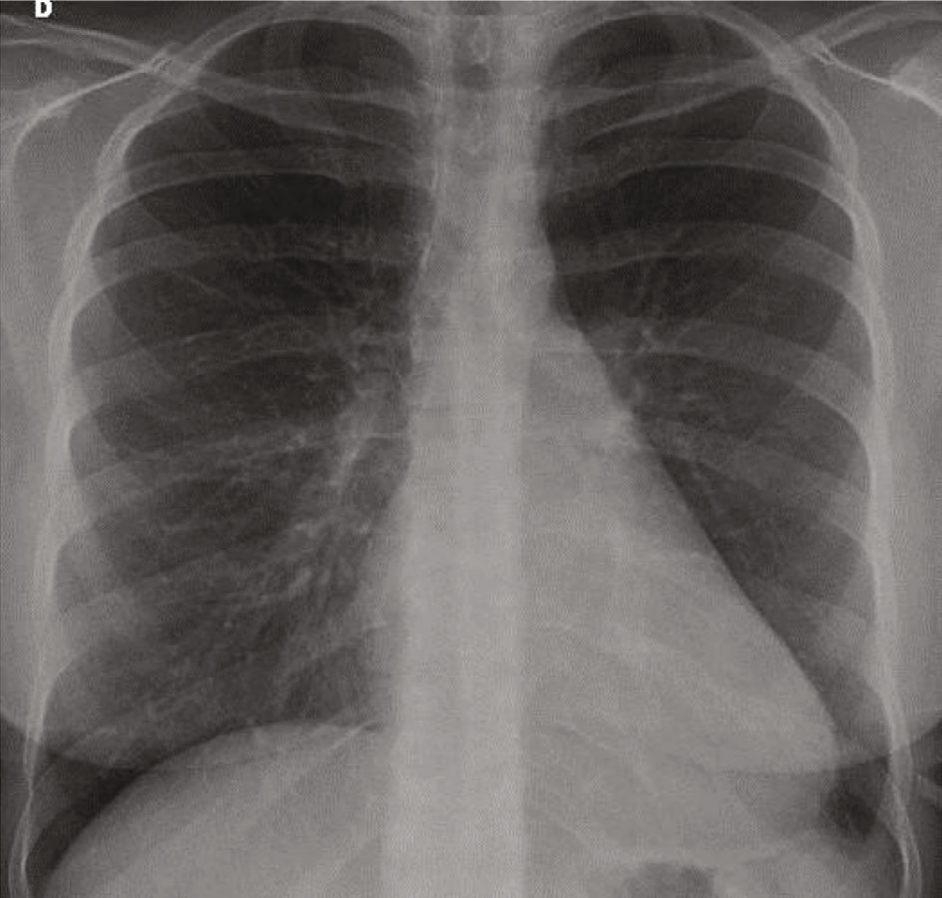
Chest X-ray showing enlargement of cardiac silhouette.

**Figure 2:**
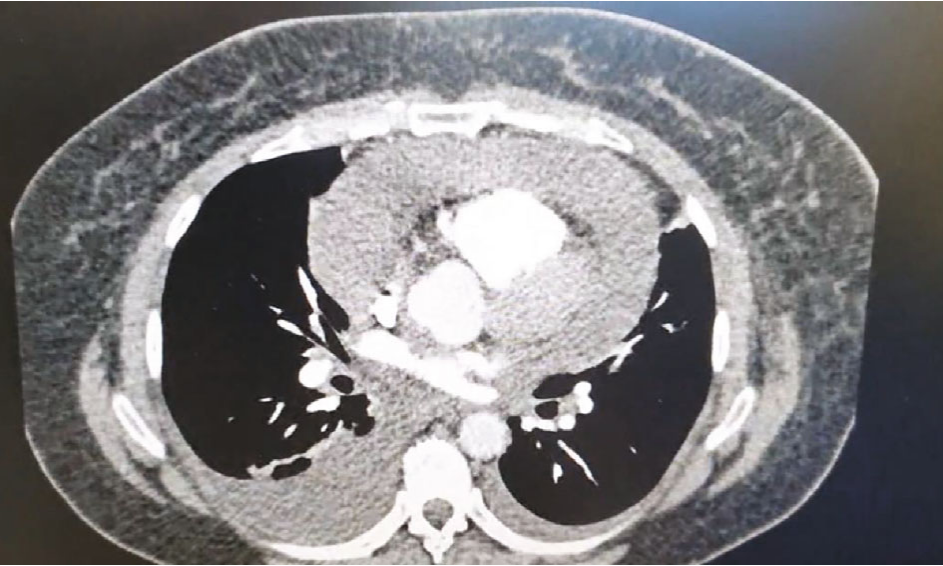
Chest TC showing pericardial effusion and pleural effusion on the right hemithorax.

A 25-year-old female patient was admitted to the emergency room complaining of dyspnea and chest pain, which persisted for the last two months. Upon physical examination on admission, the blood pressure was 120/80 mmHg and the pulse was at 100 bpm. The patient was alert, and there was no elevated jugular venous pressure, but she had muffled heart sounds and the lungs were clear on auscultation. Chest radiography presented enlargement of the cardiac silhouette (Figure 1), and electrocardiography showed low-voltage complexes. Transthoracic echocardiography demonstrated a large pericardial effusion and signs consistent with cardiac tamponade. Chest TC was performed, showing large pericardial effusion and small pleural effusion on the right hemithorax (Figure 2).

**Figure 3:**
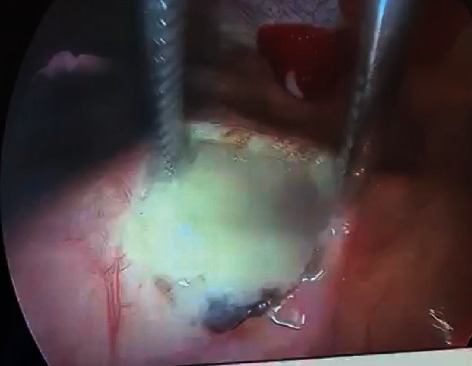
Pericardial window through VATS showing the presence of a milky fluid in the pericardium.

The patient was referred to the ICU and underwent a pericardial window through a right video-assisted thoracoscopy surgery (VATS) (Figure 3). Pericardial window showed 500 ml of a milky fluid. There were no pleural remarkable findings.

During hospital stay, lymphangioscintigraphy showed no abnormal findings. Pericardial fluid examination revealed a triglyceride level of 2050 mg/dl, total protein 5.6 g/l and glucose 154 mg/dl, all values consistent with chylopericardium. The postoperative course was uneventful; the chest tube was removed on day three, and the patient was discharged on day five. After one-year follow-up, the patient was in good condition and presented no signs of recurrence.

## 3. Discussion

Chylopericardium is defined as accumulation of lymph in the pericardial sac, a condition that occurs most frequently after cardiothoracic surgery and trauma or in association with tumors. The condition is defined as an idiopathic chylopericardium when it happens without any known precipitating factor [1, 2]. The pathogenesis might be explained by damage to the thoracic duct valves and increased permeability in the lymphatic vessels associated with elevated pressure in the thoracic duct or communication between the thoracic duct and pericardial lymphatics [2]. Besides the unclear pathophysiology, recent reports identified lymphatic leakage and communication with the pericardial sac, as explanations for the etiology of the disease [2, 3]. Until 2017, 104 cases were found in the literature and among these cases, approximately, 56% were idiopathic [4].

A normal pericardial space contains 25-30 ml of fluid [4]. In patients with chylopericardium, the fluid has a milky yellowish appearance, a cholesterol/triglyceride ratio < 1.0, triglycerides > 500 mg/dL, lymphocyte dominant fluid cell count, and negative fluid cultures [3]. Its suspect diagnosis is made mainly by imaging exams such as echocardiography, radiography, and chest CT, in addition to noninvasive techniques, such as scintigraphy with sestamibi-99mTc, a radiopharmaceutical with high cardiac absorption [3, 5]. It can also be confirmed by ingesting triglycerides or fatty acids radiologically labeled with 131I-triolein, glycerol, which acts on the lipid absorption mechanism [3, 6].

The disorder takes place in all ages and shows no sex-related variation [2, 3]. Clinical manifestations vary from asymptomatic patients, dyspnea, cough, and fatigue [2]. A recent review about the theme collected all reported cases in literature, and the most common symptoms were dyspnea (53.9%), cough (10.8%), and palpitation (9.62%). The second major part of the patients was asymptomatic (39.42%), and the most uncommon sign was edema (1.92%). On physical examination, 22.12% of the patients presented jugular venous distention and 32.69% presented muffled heart sound [3].

The ideal treatment for PIC is still uncertain; management options include diet therapy with medium-chain triglycerides, ligation of the thoracic duct, pericardiocentesis, and pericardial window. The surgical treatment should be prioritized, even in asymptomatic patients, to prevent the progression and exacerbation of the patient's clinical condition. Conservative treatment is usually not satisfactory and presents high failure rates; therefore, it should be considered when surgery is not a possibility [2].

## 4. Conclusion

Even though PIC is a rare condition, clinicians should keep in mind its possibility as a differential diagnosis. This condition requires a rapid evaluation to prevent complications and pericardial window, which, even when presenting risk of recurrence, is an effective and safe treatment option. In case of relapse of such condition, surgical ligation of thoracic duct should be considered.

## Data Availability

The data that support the findings of this study are openly available in the databases of PubMed and SciELO, and each reference presents a direct link or DOI.
